# Asiaticoside alleviates lipopolysaccharide-induced acute lung injury by blocking Sema4D/CD72 and inhibiting mitochondrial dysfunction in RAW264.7 cell and mice

**DOI:** 10.1007/s00210-024-03091-x

**Published:** 2024-04-26

**Authors:** Jianhua Zhang, Hao Zhao, Fang Wang, Jie Zhou, Mao Li, Hua Li, Meiping Ren, Lulu Wang, Qingyi Ren, Xiaolin Zhong, Xian Jiang, Zhuo Zhang

**Affiliations:** 1 Department of Anesthesiology, The Affiliated Hospital of Southwest Medical University, Luzhou, China; 2 Department of Anesthesiology, Luzhou People’s Hospital, Luzhou, China; 3 Department of Pharmacy, The Affiliated Hospital of Traditional Chinese Medicine, Southwest Medical University, Luzhou, China; 4 School of Pharmacy, Southwest Medical University, Luzhou, China; 5 Department of Pharmacology, School of Pharmacy, Southwest Medical University, Luzhou, China; 6 Department of Gastroenterology, The Affiliated Hospital of Southwest Medical University, Luzhou, China

**Keywords:** Asiaticoside, MicroRNA-21, Sema4D, Mitochondrial dysfunction, Lipopolysaccharide-induced acute lung injury

## Abstract

Acute lung injury (ALI) is a common disease with complex pathogenesis. However, the treatment is mainly symptomatic with limited clinical options. Asiaticoside (AS), a Chinese herbal extract, has protective effects against LPS-induced ALI in mice and inhibits nitric oxide and prostaglandin E2 synthesis; however, the specific mechanism of AS in the prevention and treatment of LPS-induced ALI needs further study. Sema4D/CD72 pathway, mitochondrial dysfunction, and miRNA-21 are closely associated with inflammation. Therefore, the present study aimed to explore whether AS exerts its therapeutic effect on ALI by influencing Sema4D/CD72 pathway and mitochondrial dysfunction, restoring the balance of inflammatory factors, and influencing miRNA-21 expression. Cell and animal experiments were performed to investigate the effect of AS on ALI. Lipopolysaccharide (LPS) was used to establish the ALI model. CCK8 and flow cytometry were used to detect the cell viability and apoptosis rate. HE staining and wet-to-dry weight ratio (W/D) of lung tissue were determined. The expressions of Sema4D, CD72, NF-κB p65, Bax, Bcl2, and caspase 3 in RAW264.7 cells and lung tissues were detected by western blot, and the levels of IL-10 and IL-1β induced by LPS in supernatant of RAW264.7 cells and BALF were measured by ELISA. And the expression of miRNA-21 in cells and lung tissues was detected by fluorescence quantitative PCR. The result shows that AS treatment suppressed LPS-induced cell damage and lung injury in mice. AS treatment could alleviate the pathological changes such as inflammatory infiltration and histopathological changes in the lungs caused by LPS, and reduce the ratio of W/D. AS significantly alleviated the decrease of mitochondrial membrane potential induced by LPS, inhibited the increase of ROS production, and reduced the expression of mitochondrial fission proteins Drp1 and Fis1. The high-dose AS group significantly downregulated the expression of Sema4D, CD72, phosphorylated NF-κB p65, and apoptosis-related proteins, decreased the pro-inflammatory factor IL-1β, and enhanced the level of anti-inflammatory factor IL-10. In addition, AS promoted miRNA-21 expression. These effects inhibited apoptosis and restored the balance between anti- and pro-inflammatory factors. This represents the inaugural report elucidating the mechanism by which AS inhibits the Sema4D/CD72 signaling pathway. These findings offer novel insights into the potential application of AS in both preventing and treating ALI.

**Supplementary Information:**

The online version contains supplementary material available at 10.1007/s00210-024-03091-x.

## Introduction

Acute lung injury (ALI) can be triggered by several causes including infections. The treatment modalities are customized according to the cause. Traditional Chinese medicines, such as Asiaticoside (AS) and Sosiho-Tang, may have preventive and therapeutic effects on ALI (Qiu et al. 2015). AS is a triterpene glycoside extracted from *Centella asiatica*. Its molecular weight is 959.12, and chemical formula is C48H78O19. Triterpenes have a wide range of pharmacologic properties, including neuroprotective, cardioprotective, hepatoprotective, wound healing, anti-inflammatory, antioxidant, and antitumor properties (Bandopadhyay et al. 2023, Liu et al. 2023, Liu et al. 2021, Wang et al. 2020b, c, a). Studies have found that AS could attenuate hyperoxia-induced lung injury and effectively protect from septic lung injury induced by cecal ligation and puncture (CLP) (Dang et al. 2019, Zhang et al. 2011). In addition, Asiaticoside also has a protective effect against other lung diseases, like bleomycin-induced pulmonary fibrosis (Zhang et al. 2020). Our research group has reported the protective effects of AS against LPS-induced ALI in mice (Zhang et al. 2008). It inhibits nitric oxide and prostaglandin E2 synthesis and NF-κB activation in LPS-induced RAW264.7 monocyte/macrophage-like cells (Yun et al. 2008). However, most authors have focused on only anti-inflammatory and related signaling pathways while evaluating the protective and therapeutic effect of AS in ALI, and the other molecular mechanisms remain unexplored.

ALI has a complex pathogenesis, and mitochondrial dysfunction and related signaling pathways play an important role in ALI. Semaphorin 4D (Sema4D or CD100) is a protein (MW 150 kDa) expressed on various cells, such as T cells, and tissues, such as lung tissues. The protein is involved in various biological processes, such as platelet inactivation, stimulating angiogenesis, inflammatory response, and immune regulation (Lontos et al. 2018). Soluble Sema4D (sSema4D) has immunoregulatory functions in infectious and inflammatory diseases (Maleki et al. 2016). CD72 is a low-affinity receptor of Sema4D, and Sema4D interacts with CD72 to regulate B and T cell activation (Lu et al. 2013). The protein induces the synthesis of pro-inflammatory cytokines, tumor necrosis factor-α, and interleukin-6 (IL-6) (Yoshida et al. 2015). In addition, microRNAs (miRNAs) are involved in regulating the functions of Sema4D and CD72. The miR-595/Sema4D axis promotes the malignant development of esophageal squamous cell carcinoma (Zhou et al. 2021), and Sja-miR-71a alleviates schistosomiasis liver fibrosis by inhibiting the TGF-β1/SMAD pathway (Wang et al. 2020b, c, a). However, the relationship between Asiaticoside and Sema4D has not been reported.

Mitochondrial dysfunction affects reactive oxygen species (ROS) production and activates inflammatory signaling pathways, such as toll-like receptor and nuclear factor-κB (NF-κB) pathways, leading to lung cell apoptosis and ultimately ALI (Forrester et al. 2020, Zhan and Shen 2022). Mitochondrial dysfunction is frequently associated with mitochondrial fission, and dynamin-related protein 1 (Drp-1) and mitochondrial fission protein 1 (Fis-1) are the two important proteins involved in this process. Notably, LPS can induce Drp-1 and Fis-1 expression and mediate mitochondrial fission (Lee et al. 2004, Kim et al. 2020, Jin et al. 2021, Ihenacho et al. 2021, Egner et al. 2022).

miRNAs are other important factors involved in the regulation of inflammatory response in ALI. miRNA-155 and miRNA-23a are related to pro-inflammatory responses, whereas miRNA-146a, miRNA-135a, and miRNA-21 are related to anti-inflammatory responses (Das and Rao 2022, Tahamtan et al. 2018, Essandoh et al. 2016, O'Connell et al. 2012). Liang-Ge-San, a traditional Chinese medicine formula, attenuates LPS-induced ALI by upregulating miRNA-21 (Yang et al. 2019). Therefore, AS may exert a therapeutic effect on ALI by regulating miRNA-21.

The present study proposes that Asiaticoside may exert a protective role in acute lung injury by inhibiting Sema4D/CD72 signaling and mitigating mitochondrial dysfunction, as evidenced by the modulation of protein expression, inflammatory factors, mitochondrial potential, and apoptosis in both cellular and animal models.

## Materials and methods

### Reagents

Asiaticoside was obtained from Chengdu Push Biotechnology Co., Ltd. (Sichuan, China) and prepared in dimethyl sulfoxide (DMSO) at a stock concentration of 10 mM. Anti-Semaphorin 4D/CD100, anti-CD72, and anti-GAPDH antibody were purchased from Abcam (Cambridge, USA). Anti-Bax, anti-Bcl-2, NF-κB p65 Rabbit Polyclonal Antibody, and HRP-Goat Anti-Rabbit-IgG (H+L) conjugate were purchased from Beyotime Biotechnology (Shanghai, China). Phospho-NF-κB p65 (Ser536) antibody and caspase 3 antibody were obtained from Cell Signaling Technology (Danvers, USA). DRP1 polyclonal antibody and FIS1 antibody were purchased from Signalway Antibody (Maryland, USA). β-Actin antibody was purchased from Boster (Wuhan, China). TRIzol reagent were gained from Invitrogen (Grand Island, USA). miRNA primer of miR-21, as well as U6, was purchased from Gene Pharma (Shanghai, China). HiScript III All-in-One RT SuperMix Perfect for qPCR and ChamQ Universal SYBR qPCR Master Mix were purchased from Vazyme (Jiangsu, China). IL-10 and IL-1β ELISA kits were obtained from Fankewei (Shanghai, China). Annexin V-FITC (fluorescein isothiocyanate) apoptosis detection kit, mitochondrial membrane potential assay kit with JC-1, reactive oxygen species assay kit, BCA protein assay kit, penicillin-streptomycin solution (100×) were purchased from Beyotime Biotechnology (Shanghai, China). Cell Counting K-8 (CCK8) reagent was purchased from ApexBio Technology (Houston, USA). Fetal bovine serum (FBS) was purchased from Tianhang Biotechnology (Hangzhou, China). Lipopolysaccharide (LPS 055: B5), DMEM high glucose medium, and other reagents were obtained from Sigma-Aldrich (St. Louis, USA).

### Asiaticoside and lipopolysaccharide concentration

Asiaticoside’s molecular weight is 959.12, and chemical formula is C48H78O19 (Figure S1).The dose of Asiaticoside is used in our study as the reference (Qiu et al. 2015, Liu et al. 2023, Zhang et al. 2008).

1. Concentration preparation of Asiaticoside in cell experiment: 9.59 mg of Asiaticoside was accurately weighed and dissolved in 1 mL of DMSO; the final concentration was 10 mM, and frozen at 4 °C for later use. The final use concentration of DMSO is within 1‰.

Concentration preparation of Asiaticoside in animal experiment: accurately weigh 40 mg of Asiaticoside dissolved in 10 mL of ultrapure water to make a suspension (4 mg/mL). In the experiment, mice were given 0.1 mL/10 g by gavage, that is a dose of 40 mg/kg. The 10 and 20 mg/kg groups were diluted proportionally and then given to mice by gavage at 0.025 mL/10 g and 0.05 mL/10 g.

2. Concentration preparation of lipopolysaccharide in cell experiment: 10 mg LPS was dissolved in 10 mL of sterile ultrapure water, and the final concentration was 1 mg/mL.

Concentration preparation of lipopolysaccharide in cell experiment:10 mg LPS was dissolved in 1 mL of sterile ultrapure water at a final concentration of 10 mg/mL. Animals were given LPS at a dose of 0.1 mg/10 g (10 mg/kg), equal to 10 μL/10 g by intratracheal instillation.

### Cell culture

RAW 264.7 cell is obtained from Procell Life Science & Technology Co. Ltd. (Hubei, China), and cultured in DMEM containing 10% FBS at 37 °C, 5% CO_2_, 1% 100U/mL penicillin, and 1% 100 μg/mL streptomycin. The operations required for the cells cultivation were operated on the Clean Bench (Shanghai Boxun Industry & Commerce Co., Ltd., Shanghai, China) in accordance to the standard procedures.

### Cell Counting Kit-8

The cell viability of RAW264.7 after treatment with LPS (0.1, 1, 10, 20 mg/L) or AS (0.1, 1, 10 μM) was detected by CCK8. CCK8 reagent 10 μL of supplemental solution was added for 1 h at 37 °C humidity and 5% CO_2_. The optical density of each sample was measured using microplate reader at 450 nm (Bio-Rad, USA); all operations are performed according to the instruction.

### Flow cytometry

RAW264.7 cells (1 × 10^5^) were seeded into 6-well plates. The cells were treated with LPS or AS, the spent medium was drained, and the cells were washed twice with phosphate-buffered solution (PBS). The washed cells were disintegrated with trypsin, and a gentle tap removed the cells from the bottom of the well. The cells were then resuspended in the pre-absorbed culture medium, washed with PBS, and counted. Annexin V-FITC and propidium iodide were added. A blank hole was set, an FITC hole was set only with Annexin V-FITC, and a PI hole was set only with PI. Samples were incubated in the dark for 20 min and then subjected to flow cytometry according to the manufacturer’s instructions.

### Real-time fluorescence quantitative PCR

The total RNA of the cells or lung tissues was extracted with the TRIzol reagent according to the manufacturer’s instructions. RNA extraction protocol ensured the prevention of degradation of RNA and contamination with RNAases. Total RNA was finally dissolved in DEPC-treated water (Beyotime, China). The ratio of absorbance at 260 nm and 280 nm was used to assess the purity of and RNA. The extracted RNAs were used to prepare the reverse transcription reaction system. The thermal cycling conditions were 25 °C for 30 min, 42 °C for 30 min, 85 °C for 5 min, and conservation at 4 °C. Subsequent PCRs were performed on the ice. The PCR program included an initial pre-denaturation step at 95 °C for 3 min, followed by 40 cycles for denaturation at 95 °C for 12 s and annealing at 62 °C for 40 s. The fluorescence signals were then detected. RNA expression was normalized to that of U6. When the samples were added to the eight linked tubes, each gene for each sample was replicated in at least three repeated holes. The Ct values were suggested not to exceed 30.00. The differences in Ct values between the repeated holes were suggested not to exceed 0.50; otherwise, more repeated tests were recommended. The primer sequences were:

- miRNA-21(F): 5′-AAGCGACCGTAGCTTATCAGA-3′
- miRNA-21(R): 5′-GTCGTATCCAGTGCAGGGT-3′
- U6(F): 5′-CTCGCTTCGGCAGCACA-3′
- U6(R): 5′-AACGCTTCACGAATTTGCGT-3′

The final real-time PCR data were analyzed using the 2-ΔΔCt method.

### Measurement of mitochondria membrane potential

JC-1 dye was used to detect changes in mitochondrial membrane potential (MMP). RAW264.7 cells were cultured in 6-well plates for 24 h and then treated with LPS or AS for 24 h. The treated cells were washed twice with PBS, and cell culture medium (1 mL) containing serum and phenol red was added to each well. Approximately 1 mL of JC-1 staining solution was added to each well, and the plates were incubated at 37 °C for 20 min. JC-1 staining buffer (1×) was prepared by adding 4 mL distilled water in 1 mL JC-1 staining buffer (5×) and placed in an ice bath. The cell supernatant was removed, cells were washed twice with JC-1 staining buffer (1×), and images were obtained using a fluorescent microscope (Nikon Corporation, Tokyo, Japan). The excitation and emission wavelengths were set to 490 nm and 530 nm, respectively.

### Measurement of reactive oxygen species

An ROS assay kit was used to detect reactive oxygen species (ROS) production. The cells were cultured in 6-well plates for 24 h and then treated with LPS or LPS + AS for 24 h. Fluorescent probe DCFH-DA was diluted with serum-free culture medium (1:1000), and 1 mL of dilution was added to each well. The cells were incubated at 37 °C for 20 min. The supernatant was removed, and cells were washed with a serum-free culture medium and observed under a fluorescence microscope.

### Animals

Eight-week-old male Balb/c mice (body weight 20 ± 2 g) were procured from Chengdu Dossy Experimental Animal Co., Ltd. (Chengdu, China; SYXK (Chuan) 2023-0017). Mice were kept at the Institute of Zoology of Southwest Medical University (Luzhou, China), and adaptive patterns of mice were labeled and grouped after a week. Mice were free from disease, and they were laid on clean bedding at any time, fed with sufficient water and food, and kept in a 12-h/12-h light/dark cycle. This study was conducted in accordance with the recommendations of the National Health Association Guidelines for the Care and Use of Laboratory Animals, and the protocol (SWMU20230043) was approved by the Animal Care and Utilization Committee of Southwest Medical University. Animals were randomly divided into 5 groups (sham, LPS, LPS + 10 mg/kg AS, LPS + 20 mg/kg AS, LPS + 40 mg/kg AS, *N* = 6 each group). The three AS dose groups were given 10, 20, and 40 mg/kg/day of AS by intragastric administration for 7 days before establishing the ALI model. All surgeries were performed under sodium pentobarbital anesthesia to minimize pain.

### Establishing the ALI model

The ALI model was established according to the methods in previous reports (Zhu et al. 2018). The mice were anesthetized with 50 mg/kg sodium pentobarbital by intraperitoneal injection (Zhu et al. 2018). A nasal catheter was inserted through the mouth into the bronchus and connected to a syringe for infusion. The LPS and AS groups were administered LPS (10 mg/kg) by intratracheal instillation, and the sham group was treated with an equal amount of sterile ultrapure water. Mice were sacrificed 24 h later.

### Bronchoalveolar lavage fluid collection

The mice were sacrificed under anesthesia with 50 mg/kg sodium pentobarbital, exposed mice on the operating table; after tracheal bronchial catheter inserted into the side, the other side of bronchus was ligated, 0.5 mL sterilized PBS lavage and perfusion each time, a total of 3 times, aspirate with a syringe pump for many times to obtain bronchoalveolar lavage fluid (BALF). BALF was collected in a 5-mL centrifuge tube and at 4 ℃, 1500 g × 10 min; the supernatant was collected and stored in the refrigerator at − 80 °C.

### Hematoxylin and eosin staining

Mice were anesthetized with 1% pentobarbital sodium (50 mg/kg; i.p.), and the chest cavity was fully exposed. The right lower lobe of the lung was placed in 4% fixative (BioSharp, Hefei, China) for 48 h. The fixed lung tissue was wrapped in paraffin, sliced into 5-µm thick sections, stained with hematoxylin and eosin (H&E), and observed under a light microscope (200× magnification; BX-50 Olympus (Tokyo, Japan)).

### Lung wet/dry weight ratio

After anesthetizing, the right lung of mice was obtained and blotted with absorbent paper and weighed on an electronic balance, and the lung wet weight (W) was recorded. The lung tissue was then placed in a 70 °C incubator to dry for 48 h and then weighed again, which was the dry weight (D). Lung wet/dry weight (W/D) ratio = W / D.

### Western blotting

Western blotting was used to detect the concentration of Sema4D, CD72, Bcl-2, Bax, caspase 3, Drp1, Fis1, NF-κB p65, Phospho-NF-κB p65, and GAPDH of RAW264.7 cell or lung tissue. After obtaining these proteins, it was performed with the BCA test kit. Separation was performed on a 10% or 12% SDS polyacrylamide gel and transferred to PVDF membrane (Millipore, Ameica), which was mixed with Sema4D (1:1000), CD72 (1:500), NF-κB p65 (1:1000), Phospho-NF-κB p65 (1:1000), Drp1 (1:1000), Fis1 (1:1000), Bcl-2 (1:1000), Bax (1:1000), caspase 3 (1:1000), and GAPDH (1:10,000), respectively, at 4 °C overnight, and incubated with Goat anti-Rabbit IgG antibody HRP conjugated (1:1000) at room temperature for 1 h, then washed 3 times for 10 min, and visualized by ECL reagents, the bands were analyzed by ImageJ software 6.0.

### Enzyme-linked immunosorbent assay

Supernatant was collected after cell treatment, and BALF was collected from anesthetized mice for detection; the concentrations of IL-1β and IL-10 of RAW264.7 cells and BALF were analyzed according to the manufacturer’s instructions.

### Statistical analysis

All data were analyzed with Student’s *t* test or one-way ANOVA (SNK method was used for comparison between groups). The results were presented as mean ± SD. This paper uses SPSS22.0 and GraphPad Prism 9 software to analyze all the data. *P* < 0.05 was considered to be a statistically significant difference.

## Results

### AS alleviates LPS-induced injury in RAW264.7 cells and mouse lungs

**Fig. 1:**
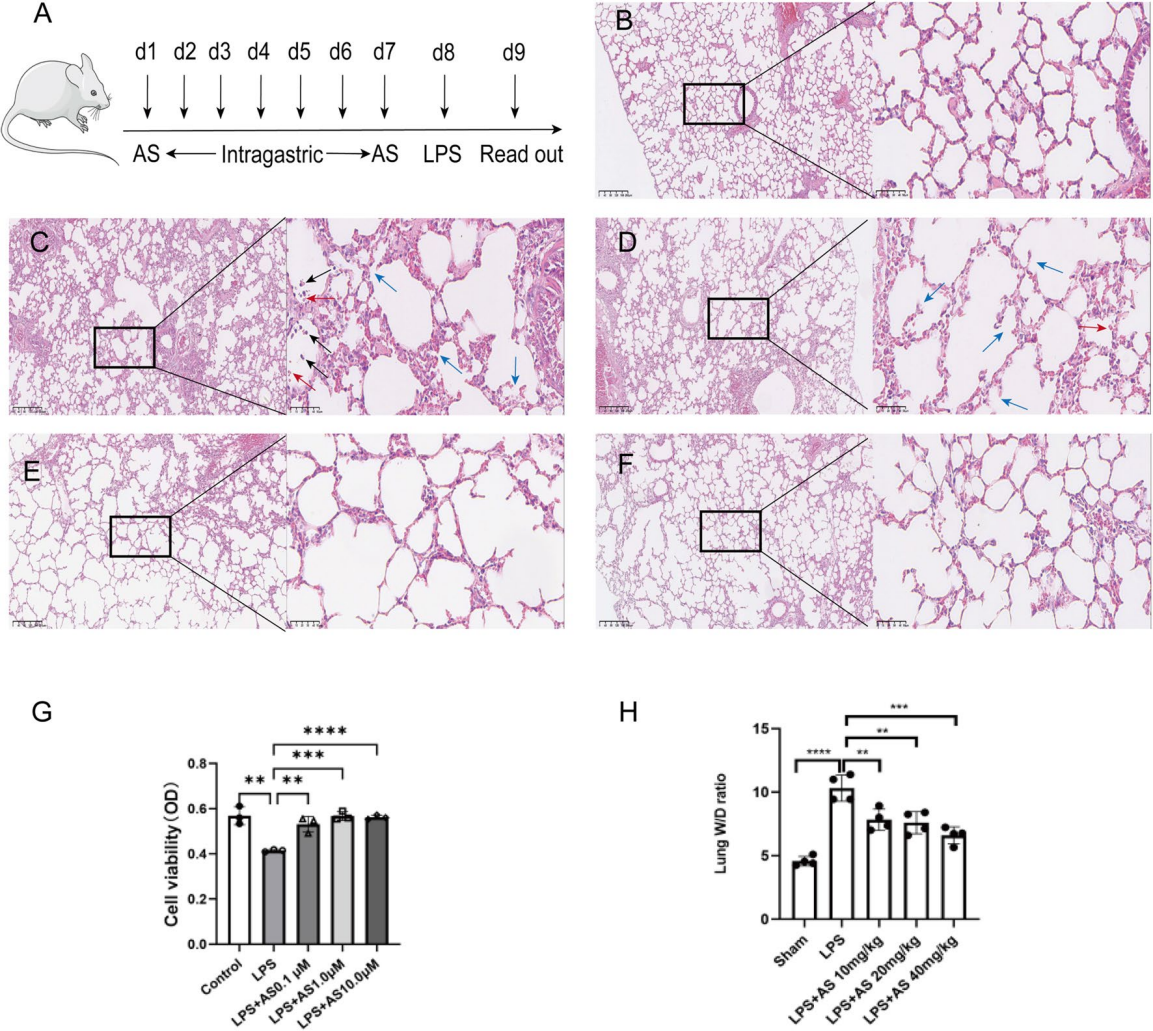
Asiaticoside alleviates RAW264.7 cell damage and mouse lung injury induced by LPS. RAW264.7 cells were randomly divided into five groups. The control group comprised normally cultured cells without LPS; the LPS group was treated with 10 μg/mL LPS; the LPS + AS groups were treated with AS (0.1, 1, and 10 μmol/L) and LPS (10 μg/mL) for 24 h. Thirty mice were randomly into five groups. The sham group included normally bred mice; the LPS group was treated with LPS, and the LPS + AS groups were intragastrically administered AS (10, 20, and 40 mg/kg/day) for 7 consecutive days. The ALI model was established in all groups except the sham group. The lung wet/dry weight ratio was determined after 24-h LPS treatment. The pathologic changes in mouse lungs were detected using H&E staining. **A** A timeline diagram of animal experiments. **B** Lung pathologic changes in the sham group (×100 and ×400). **C** Lung pathologic changes in the LPS group (×100 and ×400). **D** Lung pathologic changes in the LPS + 10 mg/kg AS group (×100 and ×400). **E** Lung pathologic changes in the LPS + 20 mg/kg AS group (×100 and ×400). **F** Lung pathologic changes in the LPS + 40 mg/kg AS group (×100 and ×400). **G** Effects of AS on the viability of RAW264.7 cells. **H** Effects of AS on the lung wet/dry weight ratio. The different colored arrows in the diagram point to exuding white blood cells (black), red blood cells (red), and broken alveolar structures (blue). Data are represented as the mean ± SD of three independent experiments. *P < 0.05, **P < 0.01, ***P < 0.001, and ****P < 0.0001, compared with the LPS group. AS, Asiaticoside; LPS, lipopolysaccharide

Lung histologic changes were determined using H&E staining. Compared with the LPS group, the AS group showed a reduction in the infiltration of inflammatory cells in the pulmonary interstitium and alveolar space and a decrease in thickening and congestion of the alveolar walls (Fig. 1B–F). Pulmonary edema is the main manifestation of lung injury, and the lung W/D ratio is the main index reflecting lung injury. The lung W/D ratio was significantly higher in the LPS group than that in the sham group (*P* < 0.01). Compared with the LPS group, the lung W/D ratio was significantly decreased in the AS groups *(P* < 0.01). These results indicated that AS protected mice from LPS-induced lung injury (Fig. 1H). The viability of RAW264.7 cells was detected using the CCK8 assay. The cell viability of the LPS group was lower than that of the control group (*P* < 0.0001). The AS groups showed significantly improved cell survival rates and alleviation of cell damage compared with the LPS group (*P* < 0.01), indicating that AS can improve cell viability after LPS injury (Fig. 1G).

### AS promotes LPS-induced miRNA-21 expression in RAW264.7 cells and mouse lungs

**Fig. 2:**
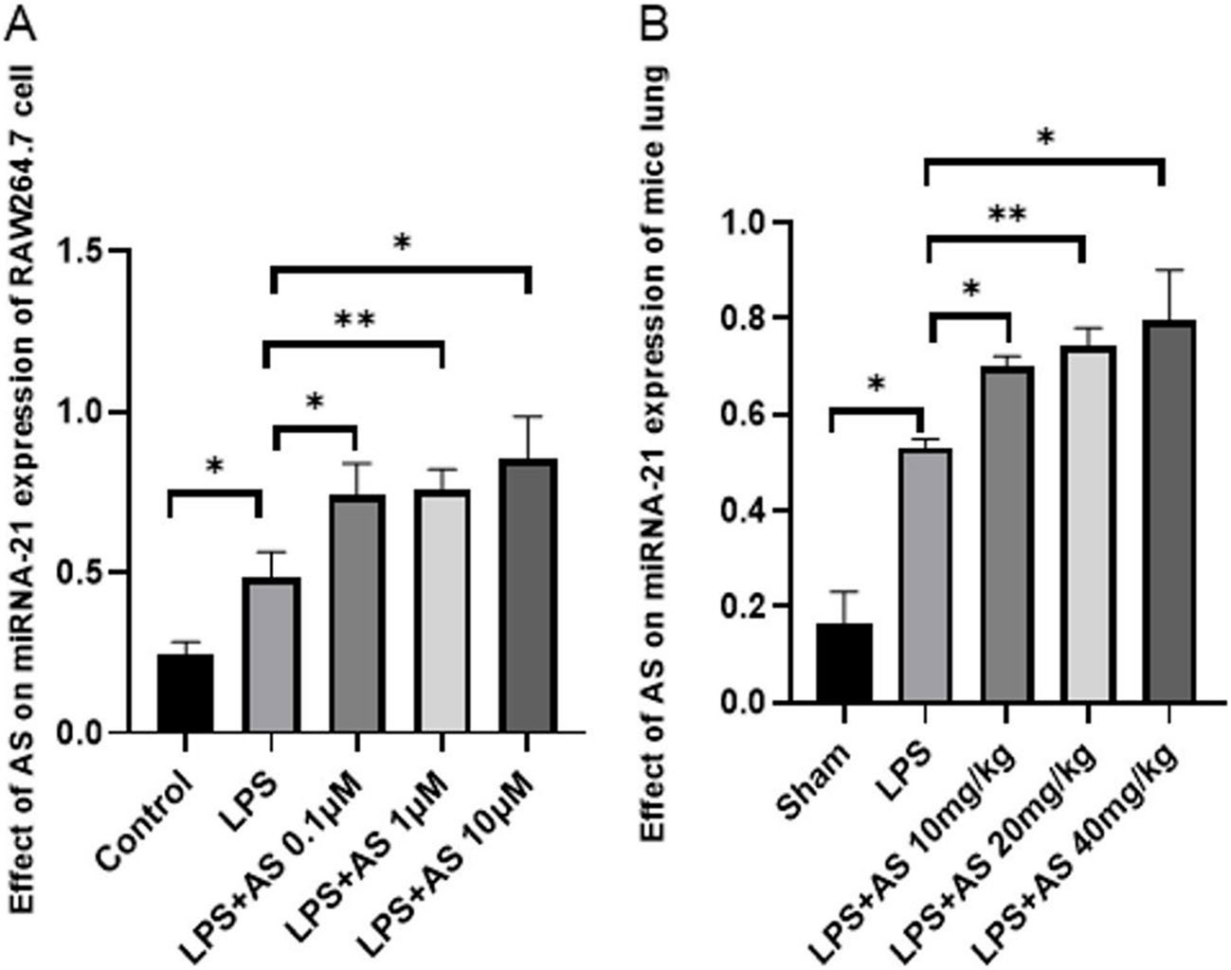
Asiaticoside promotes miRNA-21 expression induced by LPS. The relative expression of miRNA-21 in the RAW264.7 cells and lung was detected via the real-time fluorescence quantitative PCR RT-qPCR. **A** Effects of AS on miRNA-21 expression of RAW264.7 cells induced by LPS. **B** Effects of AS on miRNA-21 expression of lung induced by LPS. Data are represented as the mean ± SEM of three independent experiments. *P<0.05, **P<0.01, compared with LPS group. AS, Asiaticoside; LPS, lipopolysaccharide

The relative and absolute expression of miRNA-21 was determined using qRT-PCR to verify whether AS affects miRNA-21 expression. The expression of miRNA-21 in RAW264.7 cells was significantly higher in the LPS group compared with that in the control group (*P* < 0.05). Notably, similar results were observed in mice (*P* < 0.05). The expression level of miRNA-21 in the AS group was significantly higher than that in the LPS group in vivo and in vitro (*P* < 0.05). Overall, these results suggested that AS can increase the expression of miRNA-21 (Fig. 2A, B).

### AS ameliorates LPS-induced mitochondrial dysfunction

**Fig. 3:**
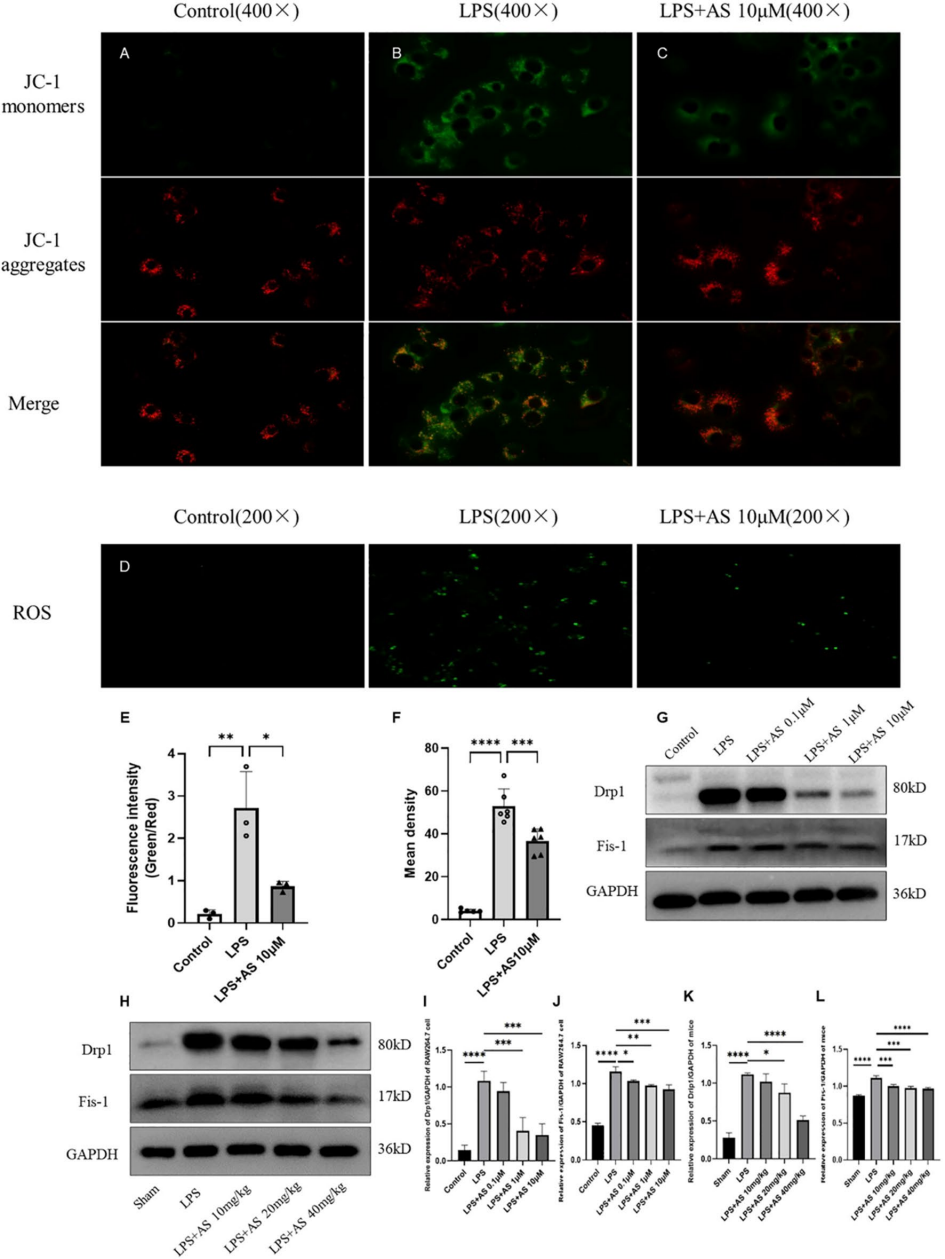
Asiaticoside inhibits LPS-induced mitochondrial dysfunction. **A** JC-1 fluorescence staining of the control group (×400). **B** JC-1 fluorescence staining of the LPS group (×400). **C** JC-1 fluorescence staining of the LPS + 10 μmol/L AS group (×400). **D** Effects of AS on ROS expression. **E** Fluorescence values indicating the effects of AS on MMP. **F** Fluorescence values indicating the effects of AS on ROS. **G** Protein bands of Drp-1 and Fis-1 isolated from RAW264.7 cells. **H** Protein bands of Drp-1 and Fis-1 isolated from mouse lungs. **I** Ratio of Drp-1 to GAPDH in RAW264.7 cells. **J** Ratio of Fis-1 to GAPDH in RAW264.7 cells. **K** Ratio of Drp-1 to GAPDH in mouse lungs. **L** Ratio of Fis-1 to GAPDH in mouse lungs. Data are represented as the mean ± SD of three independent experiments. *P < 0.05, **P < 0.01, ***P < 0.001, and ****P < 0.0001, compared with the LPS group. AS, Asiaticoside; LPS, lipopolysaccharide; ROS, reactive oxygen species; MMP, mitochondrial membrane potential

Mitochondrial membrane potential (MMP) changes, ROS generation, and mitochondrial fission reflect mitochondrial function. MMP was determined using JC-1 fluorescence staining. When the MMP is high, JC-1 accumulates in the matrix of mitochondria, forming a polymer (J-aggregates), which has bright red fluorescence and weak green fluorescence. When the MMP decreased, JC-1 could not exist in the mitochondrial inner membrane in the form of polymer, so the red fluorescence intensity decreased significantly, while the green fluorescence increased significantly. MMP was high in normal cells and decreases in apoptotic cells. The green fluorescence in the LPS group was significantly brighter compared with the other groups. Compared with that in the LPS group, the fluorescence in the 10 μM AS group was darker (*P* < 0.05), suggesting that AS can restore MMP (Fig. 3A–C, E). ROS production was detected using fluorescence staining. Compared with the LPS group, ROS production was significantly lower in the 10 μM AS group (*P* < 0.001), suggesting that AS can ameliorate mitochondrial dysfunction (Fig. 3D, F). Drp-1 and Fis-1 expression in RAW264.7 cells and mouse lungs was detected using western blotting. AS could significantly reduce the expression of Drp-1 (except 0.1 μM AS in vitro and 10 mg/kg AS in vivo; *P* < 0.001) in the LPS-treated groups. AS could significantly reduce the expression of Fis-1 in the LPS-treated groups (*P* < 0.05). Overall, these results indicated that AS can alleviate mitochondrial fission (Fig. 3G–L).

### AS decreases LPS-induced Sema4D, CD72, and NF-κB expression

**Fig. 4:**
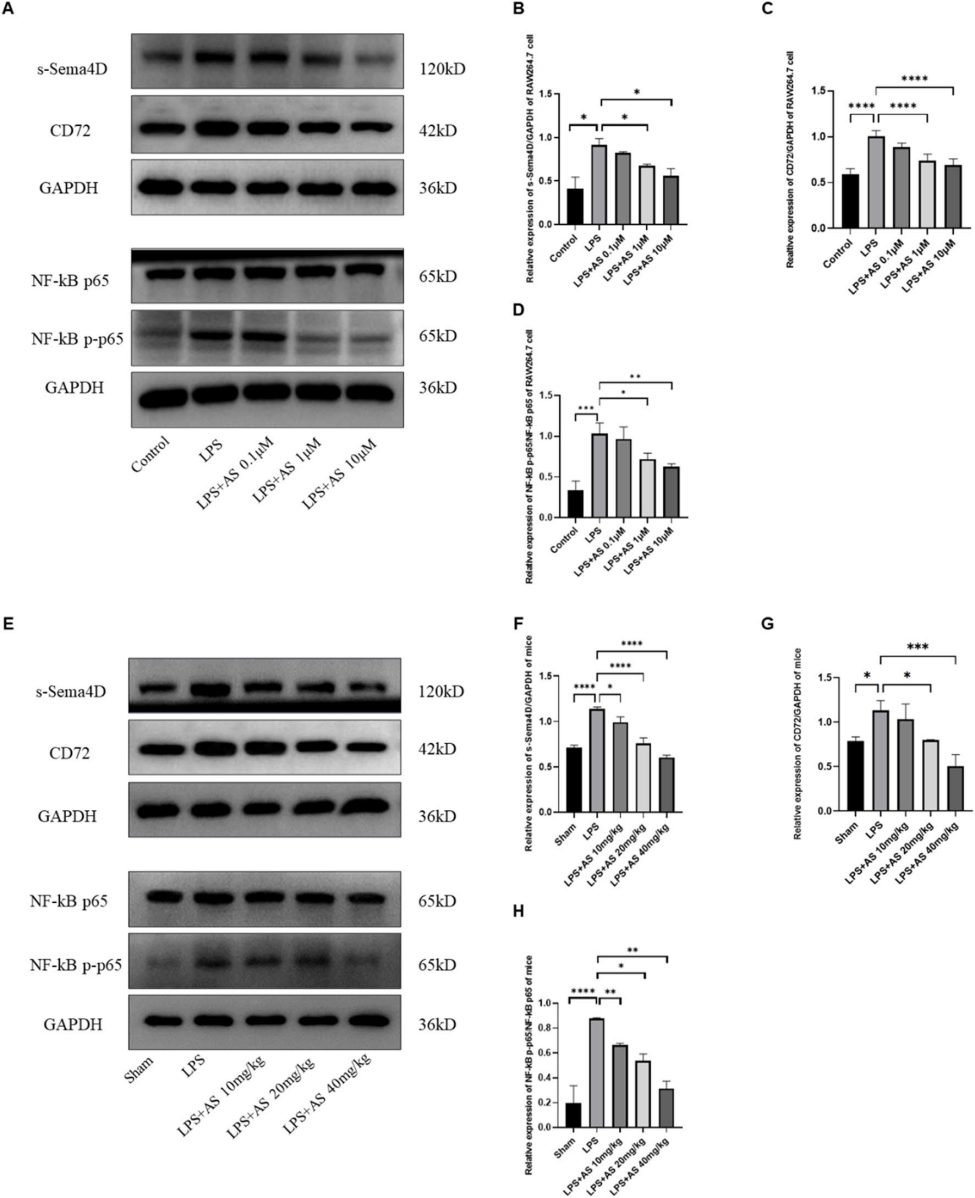
Asiaticoside inhibits LPS-induced Sema4D, CD72, and NF-κB P65 expression. Sema4D, CD72, NF-κB P65, and NF-κB pP65 expression in RAW264.7 cells and mouse lung tissues was detected using western blotting. **A** Protein bands of Sema4D, CD72, NF-κB P65, and NF-κB p-P65 extracted from RAW264.7 cells. **B** Ratio of Sema4D to GAPDH in RAW264.7 cells. **C** Ratio of s-Sema4D to GAPDH in RAW264.7 cells. **D** Ratio of CD72 to GAPDH in RAW264.7 cells. **E** Ratio of NF-κB p-P65 to NF-κB P65 in RAW264.7 cells. **F** Protein bands of Sema4D, CD72, NF-κB P65, and NF-κB p-P65 in mouse lung tissues. **G** Ratio of Sema4D to GAPDH in mouse lung tissues. **H** Ratio of s-Sema4D to GAPDH in mouse lung tissues. **I** Ratio of CD72 to GAPDH in mouse lung tissues. **J** Ratio of NF-κB p-P65 to NF-κB P65 in mouse lung tissues. Data are represented as the mean ± SD of three independent experiments. *P < 0.05, **P < 0.01, ***P < 0.001, and ****P < 0.0001, compared with the LPS group

Sema4D, CD72, and NF-κB expression was detected in RAW264.7 cells and mouse lungs using western blotting. sSema4D levels significantly increased after LPS stimulation, indicating that LPS converted Sema4D into sSema4D. High doses of AS (10 μM in vitro and 40 mg/kg in vivo) significantly decreased the production of sSema4D in the LPS + AS group compared with the LPS group (*P* < 0.05). CD72 expression was significantly increased after LPS stimulation, and AS treatment ameliorated the LPS-induced increase in CD72 expression (*P* < 0.05). In addition, AS could significantly reduce the expression of NF-κB p-p65 and decrease the NF-κB p-p65/NF-κB p65 ratio. Overall, AS alleviated LPS-induced cell damage by modulating the Sema4D/CD72/NF-κB signaling pathway (Fig. 4A–H).

### AS ameliorated LPS-induced apoptosis of RAW264.7 cells

**Fig. 5:**
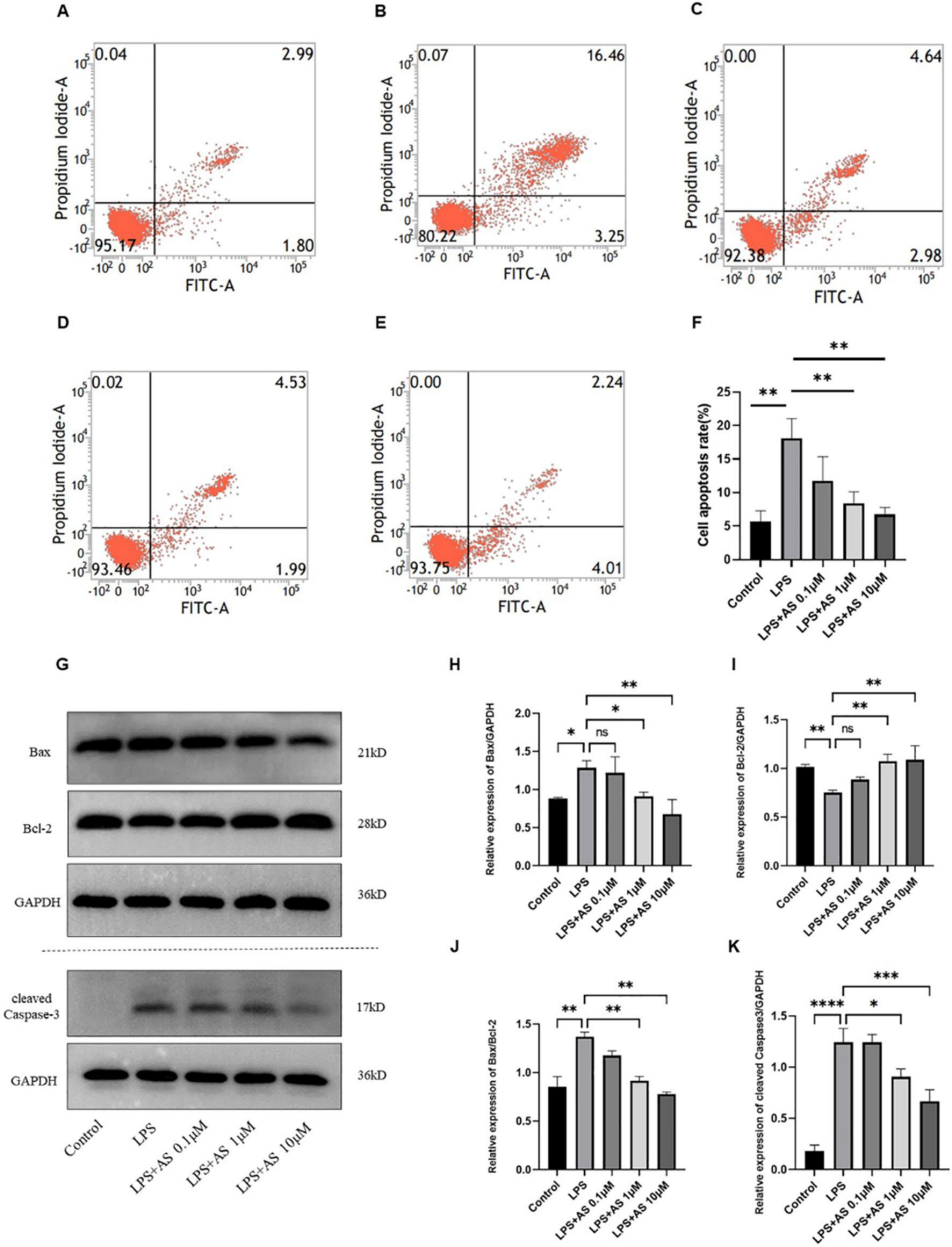
Asiaticoside alleviates LPS-induced apoptosis of RAW264.7 cells. RAW264.7 cells were randomly separated into five groups. Control group included normally cultured cells without LPS; the LPS group was treated with 10 µg/mL LPS, and LPS + AS groups were treated with AS (0.1, 1, and 10 μmol/L) and LPS (10 μg/mL) for 24 h. Cell apoptosis was detected using flow cytometry. The results of flow cytometry for **A** control, **B** LPS, **C** LPS + 0.1 μM AS, **D** LPS + 1 μM AS, and **E** LPS + 10 μM AS groups. **F** Effects of AS on the rate of cell apoptosis. **G** Protein bands of cleaved-caspase 3, Bax, and Bcl-2. **H** Ratio of Bax to GAPDH. **I** Ratio of Bcl-2 to GAPDH. **J** Ratio of Bax/Bcl-2 in. **K** Ratio of cleaved-caspase 3 to GAPDH in RAW264.7 cells. Data are represented as the mean ± SD of three independent experiments. *P < 0.05, **P < 0.01, ***P < 0.001, and ****P < 0.0001, compared with the LPS group. AS, Asiaticoside; LPS, lipopolysaccharide

The apoptosis of LPS-induced RAW264.7 cells was detected using flow cytometry. Compared with the control group, apoptosis was significantly increased in the LPS group (*P* < 0.01). Notably, 0.1 μM of AS had no inhibitory effect on apoptosis induced by LPS, and 1 and 10 μM AS had a significant inhibitory effect on apoptosis induced by LPS (*P* < 0.01; Fig. 5A–F). Caspase 3, Bax, and Bcl-2 are the three apoptosis-related proteins. Bax contributes to the progression of apoptosis, whereas Bcl-2 inhibits apoptosis. In addition, the ratio of Bax to Bcl-2 reflects the status of cell survival in response to an apoptotic stimulus. The expression of Bcl-2 was significantly decreased, and that of Bax was significantly increased in the LPS group compared with the control group (*P* < 0.01). In addition, the ratio of Bax to Bcl-2 and cleaved caspase 3 increased in the LPS group. However, 1 and 10 μM AS decreased the expression of caspase 3, Bax, and the ratio of Bax to Bcl-2 and increased the expression of Bcl-2 in LPS-treated cells (*P* < 0.05–0.001; Fig. 5G–K).

### AS restores the balance of inflammatory cytokines dysregulated by LPS

**Fig. 6:**
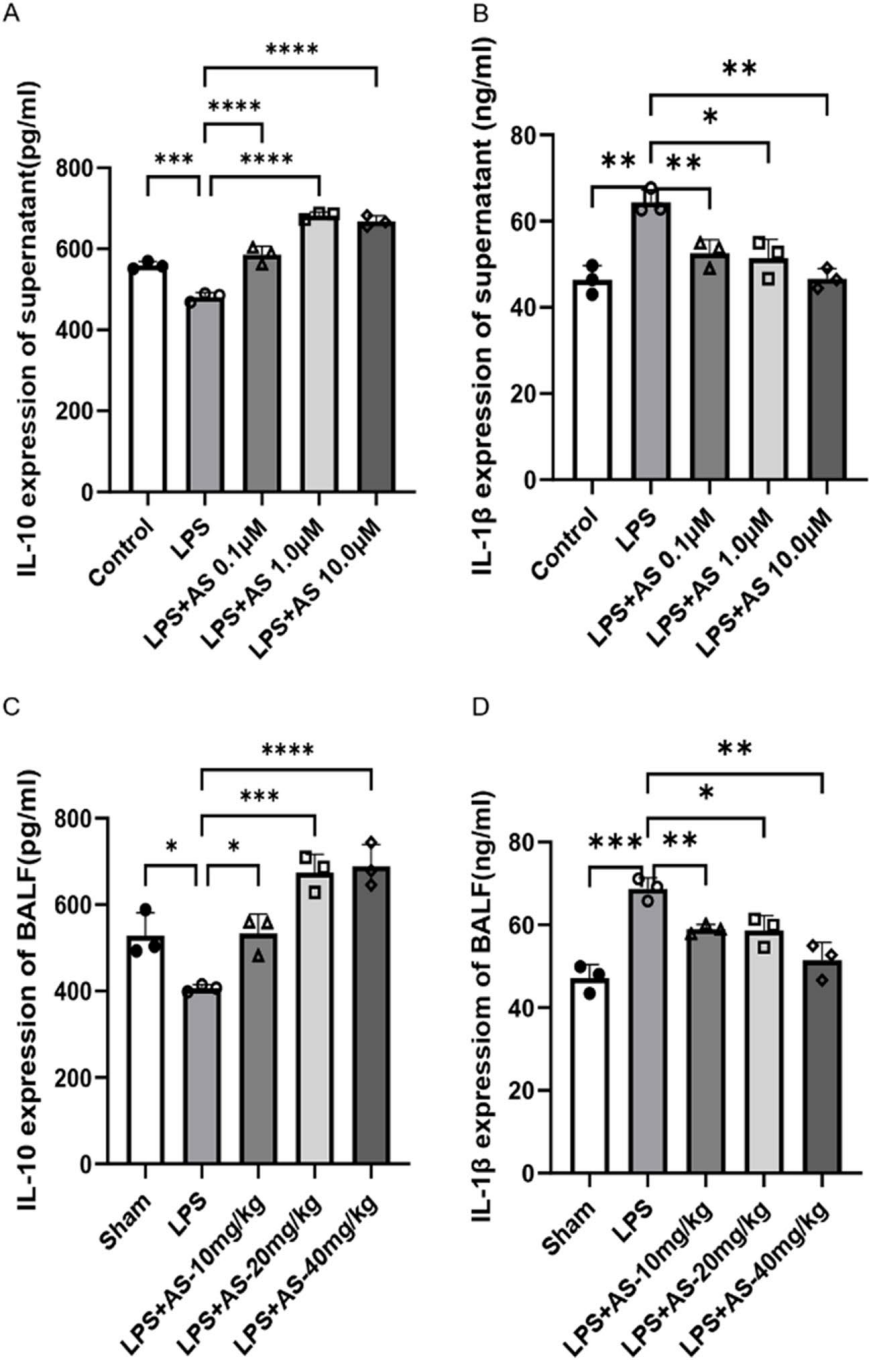
Asiaticoside promotes the expression of anti-inflammatory factors and inhibits the expression of pro-inflammatory factors to restore the balance of inflammatory factors dysregulated by LPS. ELISA-based quantification of IL-10 and IL-1β in RAW264.7 cell culture supernatant and BALF. **A** Effects of AS on the expression of IL-10 in RAW264.7 cell culture supernatant. **B** Effects of AS on the expression of IL-1β in RAW264.7 cell culture supernatant. **C** Effects of AS on the expression of IL-10 in BALF of mice. **D** Effects of AS on the expression of IL-1β in BALF of mice. Data are represented as the mean ± SD of three independent experiments. *P < 0.05, **P < 0.01, ***P < 0.001, and ****P < 0.0001, compared with the LPS group. AS, Asiaticoside; LPS, lipopolysaccharide; BALF, bronchoalveolar lavage fluid

We analyzed IL-1β and IL-10 expression in the supernatant of cultured RAW264.7 cells and BALF of mice to investigate the effect of AS on the release of pro- and anti-inflammatory cytokines induced by LPS. IL-1β expression significantly increased in the LPS group compared with the control or sham group (*P* < 0.05). The AS group showed significantly lower IL-1β expression compared with the LPS group (*P* < 0.05). In contrast, IL-10 expression was significantly lower in the LPS group compared with the control or sham group (*P* < 0.05). The AS group showed significantly higher IL-10 expression compared with the LPS group (*P* < 0.05). Therefore, AS can inhibit the expression of pro-inflammatory factors and promote the expression of anti-inflammatory factors (Fig. 6A–D).

## Discussion

ALI is one of the leading causes of death in the intensive care unit. However, only symptomatic treatment is available due to the complex pathogenesis of ALI. Traditional Chinese medicinal compounds can be used for the treatment of ALI. Dehydroandrographolide may be a potential therapeutic drug for ALI induced by coronavirus disease 19 by inhibiting the Akt/Nrf2 pathway (Pu et al. 2023). In addition, some monomeric compounds, such as AS, fusu, and punicalagin, have a certain protective effect on ALI (Dong et al. 2023, Gao et al. 2018, Zeng et al. 2022). AS can significantly inhibit LPS-induced oxidative stress, inflammation, and apoptosis. It has a protective effect on LPS-induced ALI (Qiu et al. 2015, Zhang et al. 2011, Tan et al. 2021). Our research group has reported that AS can regulate inflammatory factors in LPS-induced ALI; however, the mechanism remains unclear (Zhang et al. 2008), and needs to be further studied.

In this study, we first determined the protective effect of AS on LPS-induced cell injury and mouse lung injury. RAW 264.7 cells are derived from a tumor induced by the murine leukemia virus and are commonly used to evaluate inflammatory responses. Therefore, cell viability was evaluated in RAW264.7 cells, and pulmonary pathologic changes and lung W/D ratios were observed in a mouse model. AS increased cell viability, ameliorated the increase in the lung W/D ratio, and significantly alleviated pathologic variations (specifically at a high dose of 40 mg/kg). These findings indicate the protective effect of AS on LPS-induced pulmonary injury.

Subsequently, we explored the effect of AS on miRNA-21 in LPS-induced ALI. miRNAs, such as miRNA-34b and miRNA-155, are associated with the occurrence of ALI and are involved in its pathogenesis by regulating NF-κB and the release of inflammatory factors (Xie et al. 2018, Jiang et al. 2019, Wu et al. 2018). miRNA-21 expression is upregulated after LPS stimulation (Barnett et al. 2016). Interestingly, overexpression of miRNA-21-5p alleviates hypoxia-induced acute lung damage (Liu et al. 2019), inhibits the release of pro-inflammatory cytokines, promotes the release of anti-inflammatory cytokines, and negatively regulates inflammatory responses through NF-κB (Qin et al. 2018). This may occur because the immune system promotes the expression of anti-inflammatory factors, inhibits the expression of pro-inflammatory factors, and restores the inflammatory balance by upregulating miRNA-21 during the compensatory process after LPS stimulation. However, the increase in miRNA-21 expression cannot prevent the LPS-induced inflammatory response even with decompensation, leading to ALI (Zhou et al. 2018, Ge et al. 2016, Shimoni et al. 2007). Therefore, miRNA-21 can be used as a biomarker to monitor the development of ALI, and future studies may explore whether AS can alleviate ALI by regulating miRNA-21. The absolute and relative expressions of miRNA-21 in the AS group were significantly higher than those in the LPS group, suggesting that AS increases the expression of miRNA-21.

Semaphorins and their receptors play an important role in innate immune responses and acute inflammation (Kanth et al. 2021). Sema4D indirectly costimulates T cells, compromises blood–brain barrier, activates microglia, and inhibits remyelination in neurodegenerative diseases (Smith et al. 2015). In addition, it aggravates LPS-induced injury by activating the MAPK signaling pathway (Lei et al. 2020). The extracellular domains of Sema4D are cleaved by specific matrix metalloproteinases to produce 120 kDa bioactive sSema4D, which can reflect the activation status of Sema4D (Maleki et al. 2016). Sema4D activates NF-κB and other inflammatory pathways by increasing IL-1β concentration (Wang et al. 2020b, c, a). CD72, a type II transmembrane protein, is the main Sema4D receptor in immune cells (Lontos et al. 2018, Yang et al. 2011). Sema4D uses CD72 as a functional receptor and enhances the activation of B cells and dendritic cells by diminishing inhibitory signals from CD72 (Suzuki et al. 2008). We detected the expression of s-Sema4D, CD72, and NF-κB to investigate the mechanism by which AS inhibits the expression of inflammatory factor expression and found that the expression of s-Sema4D, CD72, and NF-κB was downregulated after treatment with AS. NF-κB activation leads to the imbalance of pro- and anti-inflammatory factors (Tan et al. 2021). miRNA-21 can block NF-κB activation and inhibit the inflammatory response. Therefore, we next explored the expression of inflammatory factors and found that AS decreases IL-β and increases IL-10 expression. Asiaticoside increased the level of miRNA-21 and decreased the expression of Sema4D. However, it is unclear whether elevated miRNA-21 directly acts on Sema4D. Overall, these findings suggest that AS may inhibit LPS-induced ALI by inhibiting s-Sema4D, CD72, and NF-κB activation and restoring the balance of inflammatory factors.

Mitochondrial dysfunction plays an important role in ALI, and mitochondrial fission mediates endothelial inflammation. Drp-1 is a cytoplasmic protein and is localized to the mitochondrial membrane after activation (Mendelsohn et al. 2022). LPS could induce Drp-1-mediated mitochondrial fission in the A549 cell line (Hou et al. 2021). Fis-1 is another typical mitochondrial fission-related protein (Sharma et al. 2021); structural studies of Fis1 reveal a dynamic region important for GTPase Drp1 recruitment and mitochondrial fission (Egner et al. 2022). We detected the expression of Drp1 and Fis-1; the results showed that AS can reduce the expression of Drp1 and Fis-1. ROS leads to cell injury in ALI. Mitochondrial ROS can activate inflammatory signaling pathways, such as the toll-like receptor pathway, and mediate NLR family pyrin domain containing 3 (NLRP3) inflammasome activity (Hou et al. 2021, Han et al. 2015, Kong et al. 2021). Therefore, we further analyzed the effect of AS on LPS-induced ROS expression and found that AS can reduce the production of ROS.

MMP reflects mitochondrial function, and a decrease in MMP is a landmark event in the early stage of apoptosis. We analyzed the effects of AS on MMP using JC-1 staining. The results showed that AS restored MMP and decreased LPS-induced apoptosis. We further analyzed the expression of apoptosis-related proteins, such as Bcl-2, Bax, and caspase 3, and found that AS can reduce the expression of Bax and the Bax/Bcl-2 ratio. These findings suggested that AS exerts its protective effect on LPS-induced ALI by restoring MMP and inhibiting apoptosis.

**Fig. 7:**
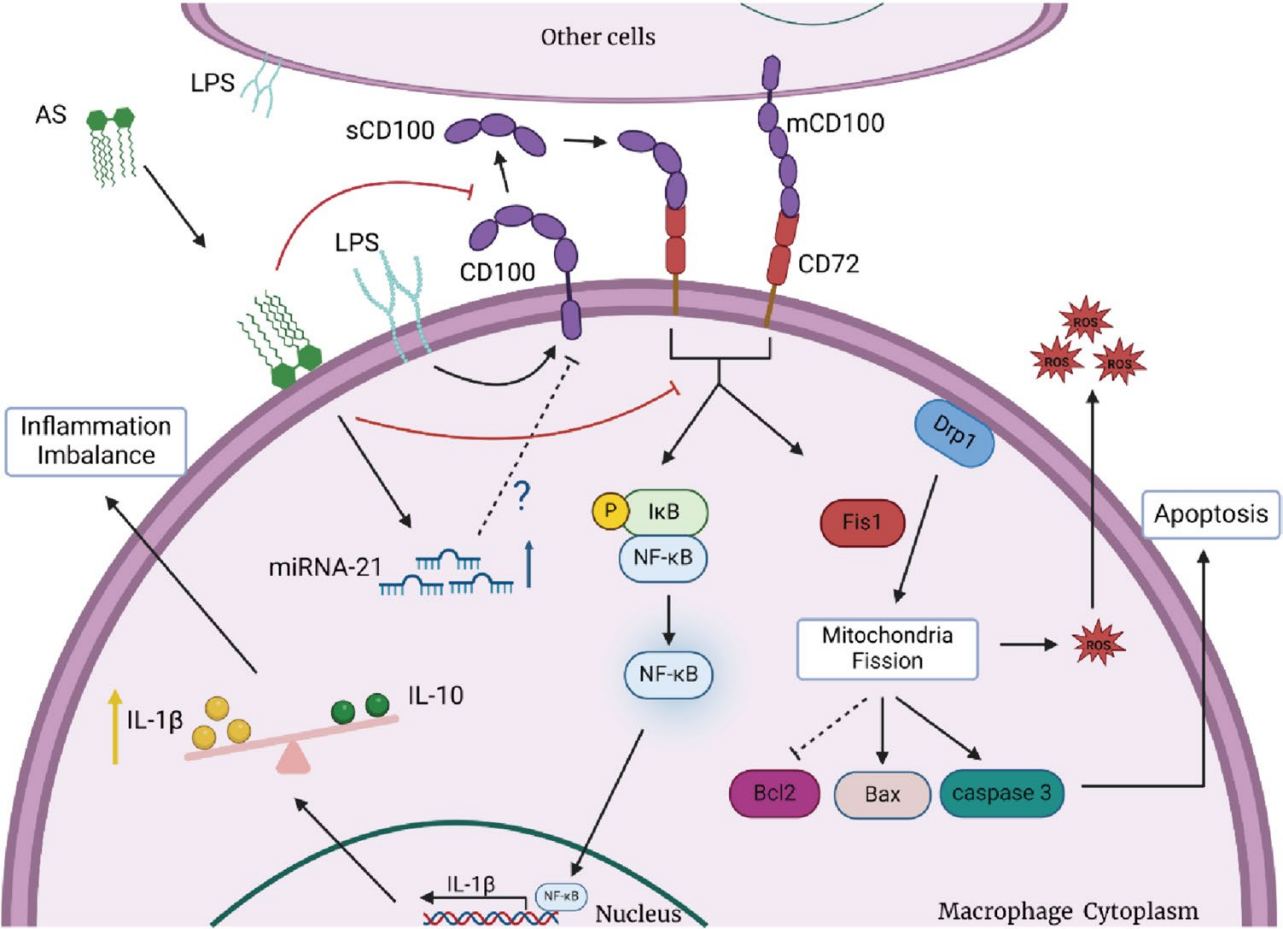
Schematic diagram of the mechanism by which AS exerts a therapeutic effect in ALI. AS may exert therapeutic effect by **A** promoting miRNA-21 expression, but the effects of miRNA-21 and Sema4D are unclear; **B** inhibiting the expression of s-Sema4D and CD72, blocking the Sema4D and CD72 interaction, inhibiting NF-κB activation, decreasing pro-inflammatory factors, increasing anti-inflammatory factors, and restoring the balance of inflammatory factors; and **C** inhibiting the expression of mitochondrial fission proteins Drp-1 and Fis-1, decreasing ROS production. In addition, AS can decrease the expression of caspase 3, Bax, and the Bax/Bcl-2 ratio, ultimately inhibiting apoptosis

Overall, AS plays a role in the prevention and treatment of ALI by promoting miRNA-21 expression, inhibiting the expression of Sema4D, and blocking the effect of Sema4D and CD72 interaction. On one hand, AS inhibited NF-κB activation by inhibiting the Sema4D/CD72 signaling pathway and the expression of pro-inflammatory factors and promoting the expression of anti-inflammatory factors, ultimately restoring the balance of inflammatory mediators. On the other hand, it inhibited the expression of mitochondrial cleavage proteins Drp-1 and Fis-1, decreased ROS production, restored MMP, and decreased the expression of caspase 3 and Bax and the Bax/Bcl-2 ratio, ultimately inhibiting apoptosis (Fig. 7). Although miRNAs, such as miR-4319 and miR-125b, can be involved in apoptosis inhibition through targeted degradation or negative regulation of Sema4D (Ren et al. 2019), it is not clear whether upregulated miRNA-21 is related to Sema4D, CD72, and NF-κB. In addition, it is still unclear whether Asiaticoside directly acts on Sema4D, and the direct or indirect relationship between Sema4D/CD72 and NF-κB is still unclear, and further experiments are needed to explore. Therefore, future studies should explore the mechanism by which miRNA-21 and Sema4D interact with each other. And further clinical trials are needed to verify the effects of Asiaticoside on ALI.

## Conclusion

AS exerts therapeutic effect in ALI by promoting miRNA-21 expression, inhibiting the Sema4D/CD72/NF-κB signaling pathway and mitochondrial dysfunction, decreasing apoptosis, and restoring the balance of inflammatory mediators.

## Supplementary Information

Below is the link to the electronic supplementary material.

Supplementary file1 (DOCX 54 KB)

## Data Availability

All data generated or analyzed during this study are included in this published article.
