# Supplementary figures and images for: Asiaticoside alleviates lipopolysaccharide-induced acute lung injury by blocking Sema4D/CD72 and inhibiting mitochondrial dysfunction in RAW264.7 cell and mice
Source: Naunyn Schmiedebergs Arch Pharmacol. 2024 Apr 26;397(10):7573–87. doi: 10.1007/s00210-024-03091-x (PMC11450039; doi:10.1007/s00210-024-03091-x)

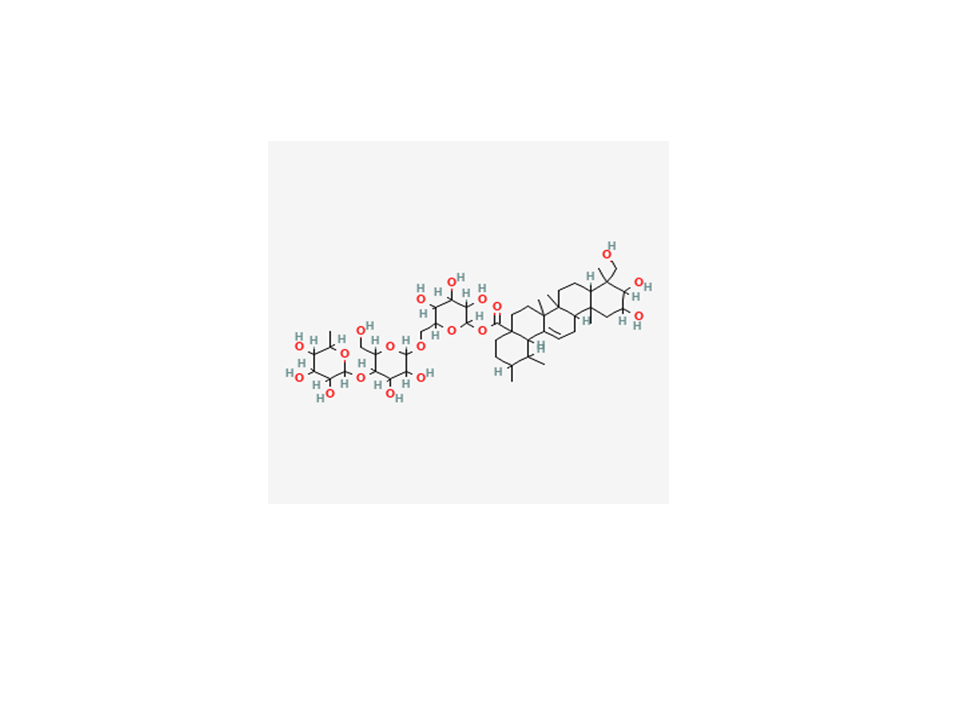


**Figure S1. Chemical structure of Asiaticoside.**

Supplement: Supplementary file 1 — Supplementary file1 (DOCX 54 KB) [file 210_2024_3091_MOESM1_ESM.docx]
